# Effects of an Avocado-based Mediterranean Diet on Serum Lipids for Secondary Prevention after Ischemic Stroke Trial (ADD-SPISE): Study protocol

**DOI:** 10.1097/MD.0000000000026425

**Published:** 2021-06-18

**Authors:** Verónica V. Olavarría, Paola Campodónico, Valeska Vollrath, Paula von Geldern, Carolina Velásquez, Patricia Pavez, Barbara Valente, Pamela Donoso, Alexandra Ginesta, Gabriel Cavada, Enrico Mazzon, Víctor Navia, Matías Guzmán, Pablo Brinck, Pablo M. Lavados

**Affiliations:** a Unidad de Neurología Vascular, Servicio de Neurología, Departamento de Neurología y Psiquiatría; b Departamento de Paciente Crítico, Clínica Alemana de Santiago; c Centro de Química Médica, Instituto de Ciencias e Innovación en Medicina, Facultad de Medicina, Clínica Alemana, Universidad del Desarrollo; d Laboratorio Clínico de Clínica Alemana de Santiago; e Escuela de Nutrición y Dietética, Universidad Mayor; f Servicio de Alimentación, Hospital Clínico Félix Bulnes Cerda; g Mutual de Seguridad y Asesorías S.A.; h Servicio de Gastroenterología, Departamento de Medicina Interna, Hospital Padre Hurtado, Servicio de Salud Metropolitano Sur Oriente; i Servicio de Gastroenterología, Departamento de Enfermedades Digestivas; j Unidad de Investigación y Ensayos Clínicos, Departamento de Desarrollo Académico e Investigación, Clínica Alemana de Santiago; k Servicio de Neurología, Departamento de Medicina Interna, Hospital Padre Hurtado, Servicio de Salud Metropolitano Sur Oriente; l Departamento de Urgencia, Clínica Alemana de Santiago, Facultad de Medicina, Clínica Alemana, Universidad del Desarrollo, Santiago, Chile.

**Keywords:** Avocado, clinical trial, diet, ischemic stroke, Mediterranean diet, secondary prevention, serum lipids

## Abstract

**Background:**

A poor or unhealthy diet is responsible for an important fraction of ischemic stroke risk. Adherence to dietary patterns, such as the Mediterranean diet, rich in monounsaturated fatty acids mainly from olive oil, is associated with a lower stroke risk. Furthermore, lowering low-density cholesterol (LDL-C) levels decreases stroke recurrence. Interestingly, Avocado-substituted diets, which are also rich in monounsaturated fatty acids, significantly decrease LDL cholesterol levels. This study aims to evaluate whether a Mediterranean diet based on Avocados reduces LDL-C compared to a low-fat high-complex carbohydrate diet after 3 months of the intervention in patients who had a recent acute ischemic stroke. The trial will also assess safety and feasibility.

**Patients and methods:**

Prospective, randomized open-label, blinded outcome assessment clinical trial. Participants are patients within a month of being admitted with an ischemic stroke, who consent and fulfil the eligibility criteria. Patients are randomly assigned to either diet intervention in a 1:1 ratio on top of the usual secondary prevention treatment. The intervention diet is:

A) Avocado-based Mediterranean diet with intake of 1/2 portion of Avocado per day and
B) The control diet is a low-fat high-complex carbohydrate diet.

The main efficacy outcome is a reduction in plasma LDL-C levels at 3 months of the dietary intervention. Secondary outcomes include changes in the levels of serum lipid profile and serum inflammation markers, safety, and feasibility. A sample size of 200 patients was estimated to provide 80% power and 5% level of significance (10% loss and 5% crossover) to detect a minimum difference of 4.6 mg/dL in LDL-C after 3 months of intervention.

**Conclusion:**

We hypothesize that an Avocado-based Mediterranean diet will further reduce the levels of LDL-cholesterol at 3 months compared to the control diet, and that the intervention is safe and feasible.

**Registration:**

The study is registered under ADDSPISE at www.clinicaltrials.gov. Identifier NCT03524742. Protocol ID CAS-605 version 3.0 (May 2nd, 2019).

## 1 Introduction

Both the Global Burden of Disease project and the INTERSTROKE study have consistently shown that a poor or unhealthy diet is responsible for an important fraction of disability-adjusted life years and population-attributable risk in ischemic stroke.^[1–3]^

Adherence to dietary patterns is associated with better cardiovascular health.^[4]^ Observational studies show that a plant based or “prudent” diet, a Dietary Approaches to Stop Hypertension diet, a Nordic diet and a Mediterranean Diet (MeDi) are associated with reduced stroke risk.^[5–8]^ Furthermore, in the PURE study, 3 to 4 serving/day of fruit, vegetable, and legume consumption was associated with a lower stroke risk.^[9]^

The “Mediterranean Diet” dietary pattern is characterized by plentiful plant foods including fruits, legumes, nuts, cereals, and olive oil as the principal source of fat rich in monounsaturated fatty acids (MUFA), moderate amounts of fish and poultry, moderate to low amounts of dairy products and low red meat, processed meats and pastries with wine consumed in low to moderate quantities usually with meals.^[10]^ A systematic review and meta-analysis showed that greater adherence to the MeDi was associated with 32% lower risk of stroke.^[11]^

Primary cardiovascular and stroke prevention with the MeDi in the PREDIMED study reported that the pooled effect of the Mediterranean diet based on olive oil or nuts was highly protective against stroke.^[12]^ Current American Heart Association guidelines suggest a Mediterranean diet after ischemic stroke (IS) with a recommendation Class IIa, Level of Evidence C, and Canadian guidelines suggest a Mediterranean diet with evidence level B, but secondary stroke prevention studies with diet are lacking and recommendations are based on extrapolations from observational or primary prevention trials.^[13–16]^

MeDi improves lipid profile, insulin sensitivity, glycemic control and decreases blood pressure, and new-onset diabetes, all strong risk factors for stroke.^[17,18]^ Furthermore, there are several beneficial nutrients abundant in the MeDi that could act on inflammatory biomarkers such as adhesion molecules, cytokines, or molecules related to the stability of atherosclerotic plaque.^[19]^ On the other hand, Avocados, although not part of the classical MeDi, are another nutrient-dense source of MUFA, rich in vitamins, minerals, fiber, phytosterols, and polyphenols. A meta-analysis of randomized clinical trials showed that Avocado-substituted diets significantly decrease the levels of total cholesterol (TC), low-density lipoprotein cholesterol (LDL-C), and triglycerides.^[20]^ A comprehensive review on the effect of Avocados concluded that they can be used as dietary supplements for the treatment of different components of the metabolic syndrome.^[21]^ No evidence exists on the effect of Avocados on stroke prevention.

Lowering LDL-C levels prevent ischemic stroke recurrence in an inverse linear relationship, without a floor effect.^[22]^ Interventions to further decrease LDL-C levels show significantly lower recurrence rates of stroke.^[23–26]^

Our primary aim is to demonstrate a reduction in LDL-C in patients who had a recent (1 month) ischemic stroke receiving a MeDi based on Avocados compared to a low-fat high-complex carbohydrate diet after 3 months. Secondary aims include the safety and feasibility of the interventions.

### 1.1 Objectives

#### 1.1.1 Primary

To show a significant LDL-C reduction in the group receiving an avocado-based MeDi (intervention diet) at 3 months in patients with IS of less than a month from symptom onset, which are receiving standard secondary prevention care, compared to controls receiving low-fat high-complex carbohydrate diet (control diet).

#### 1.1.2 Secondary

1) To show an improvement in the serum lipid profile (reduction of TC and triglycerides; increase in high density lipoprotein cholesterol [HDL-C]) in patients with IS receiving the intervention diet compared to the control diet after 3 months.
2) To investigate the change in serum inflammation markers in patients with IS receiving the intervention diet compared to the control diet after 3 months.
3) To investigate safety of the intervention diet compared to the control diet after 3 months.
4) To investigate feasibility, adherence, and acceptability of the intervention diet compared to the control diet after 3 months.

## 2 Methods

This protocol is reported according to the Standard Protocol Items: Recommendations for Intervention Trials, and the intervention is reported according to the Template for Intervention Description and Replication checklist and guide.^[27,28]^

### 2.1 Trial design

Prospective, randomized, open-label, blinded outcome assessment (PROBE design), investigator-driven, controlled superiority clinical trial.

### 2.2 Trial population and setting

Participants are patients within 1 month of an IS admitted to Clínica Alemana de Santiago, who fulfil the eligibility criteria.

### 2.3 Eligibility criteria

Patients are included if fulfilling the following inclusion criteria:

(1) Age ≥ 45 years.
(2) A recent ischemic stroke (documented cerebral infarction or transient ischemic attack within the past month prior to randomization).
(3) One or more of the following cardiovascular risk factors: hypertension, type 2 diabetes mellitus, insulin resistance, dyslipidemia (elevated LDL or total cholesterol), current tobacco use, coronary heart disease, body mass index (BMI) ≥ 25, and family history of premature cardiovascular disease.
(4) Informed consent provided.

Patients are not eligible if one or more of the following exclusion criteria are present:

(1) Comorbidities that would interfere with compliance of the interventions or low likelihood of changing dietary habits (i.e., oncological diseases under chemotherapy, institutionalized patients).
(2) Known allergy or intolerant to Avocados.
(3) Any feeding limitation that could interfere with the dietary intervention such as dysphagia.
(4) Mandatory use of drugs for other reasons that can change lipid profile (such as hormonal therapy, antiretroviral therapy, chronic steroids, etc).
(5) The possible etiology of ischemic stroke without any of the above-mentioned cardiovascular risk factors: arterial dissection, thrombophilia, cerebral vasoconstriction reversible syndrome, and other infrequent or rare causes such as vasculitis or stroke related to autoimmune diseases.
(6) Any concomitant illness with life expectancy of less than 3 months or that would interfere with the outcome assessments and/or follow-up.

### 2.4 Consent

A physician investigator invites eligible patients to participate in the trial and obtains written informed consent from the patient or legal guardian. The rational, aims, intervention, risks, benefits, and patient rights are explained in plain language. Data from patients who withdraw from the study will not be used.

### 2.5 Randomization

**Figure 1:**
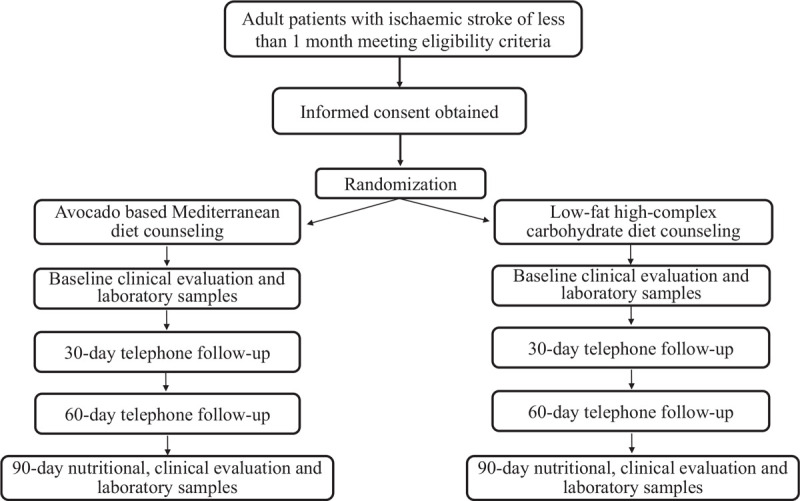
Flow diagram of the study.

Eligible patients are randomly assigned in a 1:1 ratio by study personnel to either diet intervention, using a desktop minimization program (MinimPy 0.3, Python Software Foundation), balanced by sex, age group <70 and ≥70 years (this is the median age of patients admitted with stroke in Clínica Alemana), and previous adherence to a Mediterranean diet as defined according to the Mediterranean Diet Adherence Screener (MEDAS) score <7 or ≥7 (this is the median score found in stroke patients in our institutional registry) (Fig. 1).^[29,30]^

### 2.6 Masking

Participants and investigators are not masked to the intervention diets; however, outcome assessors of LDL-C and other laboratory results, and data analysts are masked to the diet allocation. Unblinding is not anticipated.

### 2.7 Intervention

We chose a MeDi based on Avocados because of the need to have stronger evidence to support current recommendations and because avocados are widely available and used in many parts of the world more so than olive oil. We chose the low-fat control diet because it is similar to what many clinicians and dietitians still recommend and what patients do to lower cholesterol levels after stroke. We improved it using high-quality complex carbohydrates (whole grains) and fiber content.

The elaboration of diets, the content of the sessions, and written material were made and reviewed by an experienced physician nutritionist specialist and a registered dietitian nutritionist (nutritionist) with experience in Mediterranean and low-fat high-complex diets. The study nutritionist and study nurses underwent initial specific training in all study procedures by the study investigators, and subsequently all study interventions are reviewed in weekly meetings.

As part of our institutional stroke registry in all patients, a MEDAS score is obtained, which is used for randomization. The randomized diet is notified by an investigator to the study nutritionist after consent is obtained. This study nutritionist is in charge and follows the patients throughout the study.

Anthropometric measurements are performed in person by a study nurse or study nutritionist according to the study schedule (Table 1).

**Table 1:** Schedule of study evaluations.

Evaluation	Baseline	30 d	60 d	90 d
Eligibility	X			
Modified Rankin scale score	X			X
14 item Mediterranean diet questionnaire (MEDAS)	X			X
Written informed consent	X			
Randomization	X			
Secondary prevention medication questionnaire	X			X
Food frequency questionnaire	X			X
24-h food recall questionnaire		X	X	X
Rapid Assessment of Physical activity questionnaire (RAPA)	X			X
Anthropometric measures	X			X
Study blood samples	X			X
Allocated diet administration	X			
Nutritional counseling and dietary reinforcements	X	X	X	X
Questionnaire of AEs and SAEs		X	X	X
Telephone follow-up		X	X	
In person follow-up				X
Trial acceptability questionnaire				X

Several instruments are applied in a timely manner to each patient according to the study schedule outlined in the table. These included a food frequency questionnaire, a 24-hour food recall questionnaire, the Rapid Assessment of Physical Activity questionnaire, questionnaire of adverse events (AE) and serious adverse events (SAE), the MEDAS questionnaire, a structured questionnaire about the acceptability of the trial, a modified Rankin scale score, and a questionnaire about medications being administered, especially lipid lowering.

All patients received dietary counseling by a study nutritionist who explains the characteristics and composition of the allocated diet in an interview. This includes portions sizes, daily quantities, what to include and what to avoid in each diet type. Patients are also provided with detailed written information about typical foods for each dietary pattern, seasonal shopping lists, meal plans, and 14-day recipes. In all cases, the allocated diet starts when the patient is at home and before 1 month of symptom onset. The study nutritionist in charge of the patient is available through telephone during the entire participation period for any extra contact the patient may need. Patients in both intervention arms receive the same intensity of dietary counseling. Each patient receives at least 4 sessions with a study nutritionist during the 3 months of intervention.

In the baseline session, the patient receives nutritional counseling and education according to the randomized diet, applying the instruments as described in the study schedule (Table 1). The usual duration of this session is ±90 minutes. At 30 ± 7 and 60 ± 7 days, telephone follow-up is performed by the same nutritionist who inquiries about the diet pattern, applies the instruments according to the study schedule, and provides specific feedback recommendations and reinforcements on the allocated diet pattern. The usual duration of these sessions is ±30 minutes. At 90 + 14 days, follow-up a closing nutritional session is conducted including the application of the appropriate instruments according to the study schedule (Table 1), and the delivery of dietary recommendations for healthy habits as post-trial care. In this session, 1 of the physician investigators obtains the modified Rankin Score, the details of the medications being taken by the patient and details about any AE or SAE if they occurred. The usual duration of this session in ±45 minutes. Before the pandemic, the baseline and 3 months sessions were carried out in person, scheduling a visit to the hospital by the patient, since March 2020 to date these sessions are mixed: measurements and samples in person and intervention/instruments by video conference. If the patient is unable to answer, the caregiver living with the patient or who oversees cooking and feeding the patient is interviewed.

The diets are standard normocaloric adjusted depending on the nutritional status and physical activity of the patient, but the focus of both diets is on the quality of the food and not the quantity. We anticipate that weight loss may occur in some patients due to improved eating habits and food selection. For each intervention, 3 different daily caloric dietary contributions were made: 1500, 1800, and 2000 calories. The macronutrient distribution of each diet intervention is as follow:

A. Mediterranean diet: 50% total caloric value of carbohydrates, 35% fat (22% MUFA fat, 6% polyunsaturated fatty acids (PUFA) fat, ±10% saturated fat), and 15% protein.
B. Low-fat high-quality carbohydrate: 55% carbohydrates, 30% fat (12–14% MUFA fat, 6–8% PUFA fat, ±10% saturated fat), and 15% protein.

Both diets include natural foods, little processed, and naturally low in sodium, and cooking with little added salt and using spices to season meals is reinforced during food education. With these guidelines, it is intended that the patient consumes a maximum of 5 g of salt per day between foods that contains it and the added salt. In both diets, the consumption of oil is daily, and it is recommended to include it raw to dress salads. The MUFA content of the low-fat high-quality carbohydrates is 12% to 14% versus 22% in the Mediterranean diet; therefore, the selection of foods that are included in these minutes corresponds to this calculation. The contribution of olive oil to be used is lower than that in the classical Mediterranean diet and can be replaced by canola oil if the patient does not consume olive oil, and the frequency of foods rich in MUFA (nuts, avocado) and PUFA (fish) is also less than that indicated in the Mediterranean diet. Both diets include complex carbohydrates, that is, from whole grains, fruits, vegetables, legumes, meats, and low-fat dairy products, and very low or no contribution of simple sugars. In patients with diabetes mellitus, the contribution of complex carbohydrates is the same as that in non-diabetic patients. Depending on the pharmacological treatment that the patient has for diabetes mellitus, an adjustment of the number of mealtimes can be made to avoid hypoglycemia if the patient uses sulfonylureas or insulin. The meal plan includes specific recommendations for each diet intervention, as well as a menu divided into 4 meals and a snack during the day with options for 14 days. This menu must be replicated and combined during the 3 months of patient participation.

To maintain diet fidelity with the intervention, in all contacts the study nutritionist emphasizes that the portions and frequencies of food of the diet assigned to the patient are respected, solve specific doubts, and inquire about AE and SAE. The food surveys are analyzed qualitatively and quantitatively by the study nutritionist in charge of carrying them out. The qualitative analysis considers the number of meals per day, fluid intake, and quality of the food consumed, which is compared to each of the diet interventions. The quantitative analysis is carried out by obtaining the ingredients and quantities of each food preparation that the patient consumes, this is then transformed into portions to obtain the intake of calories, carbohydrates, fats, and proteins. This is compared with the requirements of the assigned diet and the % of adequacy is calculated ([intake value/requirement value] × 100). An adequate intake is considered if the macronutrient is in a percentage of adequacy of 90% to 110%. We use as a reference the contributions of nutrients and calories from the “Exchange portions and Chemical Composition of foods of the Chilean food pyramid” National Institute of Food Technologies, 1999 and its update “Food Composition Table” National Institute of Food Technologies, 2018.^[31]^ This information is used to personalize each interview and adjust counselling.

As part of the institutional protocol and Chilean stroke national guidelines, all patients receive appropriate secondary stroke prevention and are discharged with an antithrombotic statin at high doses (usually atorvastatin 80 mg day, a hypotensive, and if necessary, a hypoglycemic drug). The patients are counseled at each study visit to keep their secondary prevention medications according to their treating physician prescriptions.

### 2.8 Study outcomes

#### 2.8.1 Primary outcome

The main efficacy outcome is a reduction in the mean level of plasma LDL-C at 3 months of 4.6 mg/dL (0.3 mmol/L) or more in the intervention diet group compared to the control diet group.

#### 2.8.2 Secondary outcomes: all at 3 months

1) Levels of serum lipid profile: Significant reduction in the mean TC and triglycerides and significant increase in mean HDL-C in the intervention diet group compared to the control diet group.
2) Improvements in the levels of serum inflammation markers vascular cell adhesion molecule, Intracellular Adhesion Molecule, Interleukin-6, apolipoprotein A and B in the intervention diet group compared to the control diet group.
3) Safety: Frequency of AE and SAE by intervention group.
4) Feasibility, adherence, and acceptability:
  a) the proportion of patients who complete the intervention at 3 months and time from symptom onset to intervention as a measure of feasibility;
  b) median MEDAS score at the end of the follow-up and adequacy of the interventions as assessed by the quantitative evaluation of the food questionnaires at 30, 60, and 90 days (percentage of adequacy) to measure adherence;
  c) acceptability will be assessed at 90 days using a structured questionnaire.

### 2.9 Recruitment

Patients are recruited by physician investigators, who daily screen stroke admissions and contact attending vascular neurologist as needed to ensure patient recruitment before discharge. The neurology staff is reminded about the study during the year with a short presentation during regular clinical meetings. The recruitment objective was 6 patients per month, but the rate has been 4 per month. We expect complete recruitment by the end of 2022.

### 2.10 Measurements

Anthropometric measurements are performed by a study nurse or a study nutritionist according to the study schedule (Table 1). Weight and height are measured by using standard calibrated scales, respectively; waist circumference midway between the lowest rib and the iliac crest by using an anthropometric tape, and blood pressure after 10 minutes of rest in triplicate with a standard calibrated sphygmomanometer.

Blood samples are collected following overnight fasting for at least 10 ± 2 hours, at baseline and at 3 months, coded, centrifuged, and processed in the morning (lipids) at Clínica Alemana or stored at <80°C until assayed (for inflammatory biomarkers, see supplement) at The Medical Chemistry Center, Universidad del Desarrollo. Total cholesterol, LDL-C, HDL-C, and TG are processed with a homogeneous enzymatic colorimetric test in the chemistry automatic analyzer COBAS 8000 platform (Cobas, Roche Diagnostics) upon arrival to the laboratory. All tests are performed according to the manufacturer's instructions. The laboratory technicians are masked to the interventions. Analyses are performed for each participant in frozen samples of whole serum or plasma, as appropriate. Details of the analysis are provided in the Supplementary Material.

### 2.11 Data collection, retention, and follow-up

Data collection and follow-up assessments are performed by trained study personnel according to the schedule in the table. Patients are encouraged to stay in the study even if they are non-adherent to the assigned intervention diet and complete follow-up assessment. Extra telephone calls are performed by the study nutritionist according to the patient's needs.

### 2.12 Feasibility, adherence, and acceptability

To maintain adherence to and assess the feasibility of the intervention, participants are contacted by telephone at 1 and 2 months after recruitment. The telephone calls are made by study nutritionists to each participant to reinforce the allocated diet and administer a 24-hour diet recall questionnaire. Patients have extra telephone calls and email contacts depending on the individual evaluation of each nutritionist.

To increase adherence, randomized patients are transferred monthly an amount of money equivalent to buying avocado (2 kg per week, including family consumption) or whole grain bread (2 kg per week, including family consumption), according to the allocated diet.

The acceptability of the trial in both groups is assessed with a structured 14-item self-applied (or caregiver) questionnaire performed at the end of follow-up. Items are measured using a 5-point Likert scale.

### 2.13 Safety

Adverse events associated with the intervention diet are collected from the time of consent until the final 3-month follow-up visit for each participant, whether considered related to the intervention study or not. All adverse events are followed up until they are resolved and are adjudicated by an independent investigator. Investigators assess any adverse effects of the interventions by administering a checklist of symptoms, including mouth symptoms, bloating, fullness, indigestion, altered bowel habits, other diet-related symptoms, and any other symptoms. Serious adverse events are defined according to International Good Clinical Practice recommendations.^[32]^ Safety specifically include all cardiovascular events (acute myocardial infarction, any stroke, cardiovascular procedures, and cardiovascular deaths) and deaths. All patients are adjudicated by an independent investigator and handled by the treating physician.

### 2.14 Data quality assurance

The trial data quality is monitored by the study team. Data access is restricted to trained staff with unique password-protected accounts. No interim analyses will be performed. Independent yearly audits are performed by the Institutional Review Board and approved by the ethics committee.

### 2.15 Data management

Data is collected in paper case report forms and transferred to a Research Electronic Data Capture institutional database (REDCap Vanderbilt University) by the study personnel. Identifiable data is not recorded in the database or other documents, and participants are identified busing a unique trial ID. Documents are kept securely in a locked filing cabinet in an office, together with the signed informed consent forms that are accessible only to key research team members. Only the steering committee members and the study statistician will have access to the final trial datasets. Participant files and other source data (including copies of protocols, questionnaires, original reports of test results, correspondence, and records of informed consent) will be kept for 5 years after completion of the study.

### 2.16 Sample size estimates

The sample size was calculated using data from previous small, randomized trials of Avocado-based diets on LDL cholesterol in primary prevention, showing a mean of 131.2 (9.4) mg/dL in the standard diet group and 126.6 mg/dL (4.6) in the Avocado-diet group.^[33]^ A sample size of 100 patients per group (200 in total) was estimated to provide 80% power and 5% level of significance with 10% loss and 5% crossover to detect the same difference in LDL-C after 3 months of intervention in patients with IS.

### 2.17 Statistical analyses

All efficacy analyses will be performed according to the intention-to-treat principle. The primary outcome will be compared between the 2 groups by an analysis of covariance adjusting for baseline measures unbalanced by randomization and with baseline LDL-C and statin use (initial and final doses) as covariables. To account for missing data, multiple imputations using chained equations will be used, assuming randomness. The results will be compared to those with complete data. A sensitivity analysis will be carried out to investigate the robustness of the findings according to adherence to the intervention. Safety analysis will be performed for all patients participating in the study. The number of patients with AEs, the occurrence of specific SAEs, and discontinuation due to SAEs will be tabulated. Binary outcomes, such as AEs, major cardiovascular events (acute myocardial infarction, stroke, or vascular death), or death will be analyzed with the Chi-2 test for independence. Analysis of adherence will be performed in all patients who finalized the study. The proportion of patients who completed the intervention at 3 months will be analyzed using the Chi-2 test for independence. The percentage of adequacy of the diet intervention at 1, 2, and 3 months will be analyzed with generalized linear mixed models for repeated measures. Feasibility assessed as the mean time from symptom onset to intervention will be analyzed using Student's *t* test. Analysis of the acceptability 14-item questionnaire will be performed in all patients completing the follow-up with the Mann–Whitney–Wilcoxon non-parametric test. Exploratory subgroup analyses will be carried out irrespective of whether there is a significant treatment effect on the primary outcome. These analyses will be adjusted for confounders. The following pre-specified subgroup analyses will be performed by adding an interaction term to the models: age (< or ≥ mean) years, sex, education level < or ≥12 years, post-stroke disability (modified Rankin score < or ≥1), physical activity (Rapid Assessment of Physical Activity questionnaire score < or ≥6), MEDAS score (< or ≥ median), dyslipidemia (yes/no), diabetes/insulin resistance (yes/no), BMI (< or ≥25), and BMI (< or ≥30). Per protocol analysis will be performed for the primary and key secondary efficacy outcomes, using the same statistical test as per the intention-to-treat analysis.

The alpha error level was set to 0.05. Data will be processed in STATA (Stata version 16.0, StataCorp).

### 2.18 Ethical approval

Ethics approval was granted from Universidad del Desarrollo, Clínica Alemana de Santiago Scientific Ethics Committee, Santiago, Chile on May 23rd, 2018 (ID 2018–43), and further approved by Clínica Alemana Institutional Review Board to the first version on May 30th, 2018 (ID 605) and subsequently to versions 2 (August 14th, 2018) and 3 (May 2, 2019). Written informed consent is obtained from each participant prior to any study procedures.

### 2.19 Study organization and funding

The study is organized with a Steering Committee (VVO, PML, PC); a Committee of Clinical Events Adjudication (AG, VVO), data managers (PvG and CV), and a statistician (GC). A data monitoring committee was not deemed necessary due to the low risk of the intervention and because no interim analysis would be performed.

This study is funded by the Agencia Nacional de Investigación y Desarollo through the Fondo Nacional de Ciencia y Tecnología (FONDECYT regular N° 018/FONDECYT/882) grant number 1181333 on March 29th, 2018.

The study funders had no role in the study design; collection, management, analysis, or interpretation of data; writing of the report; and the decision to submit the report for publication, nor will they have any ultimate authority over any of these activities.

### 2.20 Status of the trial

The trial is currently randomizing patients. The first patient was randomized in August 2018, and we expect the last patient to be included in 2022.

### 2.21 Dissemination

The study results will be disseminated via publications in peer-reviewed journals and social media. Copies will be deposited at the repositories of the libraries of participating institutions. Study participants will be informed of the results by email or telephone by the study investigators.

### 2.22 Data availability statement

The specific protocol documents such as questionnaires (in Spanish) and datasets generated during the current study will be available from the corresponding author upon reasonable request.

## 3 Discussion

Currently, there is insufficient evidence to recommend a specific dietary pattern for secondary prevention in patients with IS. Several studies on diet and stroke prevention found either a protective effect of plant-based patterns against stroke or a detrimental effect of adherence to westernized patterns, but they are observational or primary prevention desings.^[34]^

There is strong evidence that a lower LDL-C level is associated with a decrease in stroke recurrence risk. The association is linear without a floor effect, so that the lower the LDL-C, the lower the stroke risk.^[35]^ Pharmacological interventions other than statins to further decrease LDL-C are a very promising venue and are being actively investigated, showing significantly lower recurrence rates, but with the caveat of a very high cost and possibility of inducing autoantibody resistance. An appropriate diet could be another way of achieving this goal, probably at a lower cost and with other beneficial metabolic and cardiovascular effects.^[20]^

An Avocado-Based Mediterranean Diet on Serum Lipids for Secondary Prevention after Ischemic Stroke Trial was designed to determine the efficacy of an Avocado-based Mediterranean diet compared to a low-fat, high-complex carbohydrate diet to lower LDL-C as a secondary stroke prevention endpoint in patients within a month of IS, and to assess the safety and feasibility of this intervention. This trial was co-designed with experienced dietitian physicians and registered dietitian nutritionists to achieve high acceptability and adherence to diet interventions in our population and to minimize bias. We selected patients older than 45 years and with at least 1 vascular risk factor to focus on a population with higher atherothrombotic risk.^[36]^

The Mediterranean diet has a high MUFA content, especially extra virgin olive oil (rich in oleic acid). Avocados are also high in MUFA and other fat-soluble vitamins. One avocado (136 g, without skin and the seed) contains approximately 13 g of oleic acid, which is similar to the amount of oleic acid in 1.5 oz (42 g) almonds or 2 tablespoons (23 g) of olive oil; thus, avocados could substitute at least part of the MUFA from olive oil in the MeDi.^[37]^

The results of An Avocado-Based Mediterranean Diet on Serum Lipids for Secondary Prevention after Ischemic Stroke Trial will address a gap in knowledge on the effect of dietary interventions on secondary stroke prevention.^[34]^ This pilot trial will help design a phase 3 clinical trial that could have an important impact on current stroke research and, ultimately, clinical practice, influencing dietary recommendations with important beneficial effects on cardiovascular outcomes.

## Author contributions

VVO and PML performed conceptualization, data curation, formal analysis, funding acquisition, investigation, methodology, project administration, resources, software, supervision, validation, visualization, writing – original draft, and writing – review and editing. PC performed conceptualization, data curation, investigation, methodology, project administration, resources, software, and writing – review and editing. PvG, PP, and CV performed data curation, investigation, project administration, supervision, validation, writing – original draft, writing – review, and editing. BV and PD performed data curation, investigation, project administration, supervision, validation, writing – original draft, writing – review, and editing. VV performed data curation, resources, validation, writing, review, and editing. AG performed validation, writing, reviewing, and editing. GC performed the formal analysis, writing – original draft, writing – review, and editing. EM, VN, MG, and PB performed data curation, investigation, and writing – review and editing.

**Conceptualization:** Veronica V. Olavarria, Paola Campodónico, Pablo M. Lavados.

**Data curation:** Veronica V. Olavarria, Paola Campodónico, Valeska Vollrath, Paula von Geldern, Carolina Velásquez, Patricia Pavez, Barbara Valente, Pamela Donoso, Enrico Mazzon, Víctor Navia, Matías Guzmán, Pablo Brinck, Pablo M. Lavados.

**Formal analysis:** Veronica V. Olavarria, Paola Campodónico, Gabriel Cavada, Pablo M. Lavados.

**Funding acquisition:** Veronica V. Olavarria, Pablo M. Lavados.

**Investigation:** Veronica V. Olavarria, Paola Campodónico, Paula von Geldern, Carolina Velásquez, Patricia Pavez, Barbara Valente, Pamela Donoso, Enrico Mazzon, Víctor Navia, Matías Guzmán, Pablo Brinck, Pablo M. Lavados.

**Methodology:** Veronica V Olavarria, Paola Campodónico, Pablo M. Lavados.

**Project administration:** Veronica V Olavarria, Paula von Geldern, Carolina Velásquez, Patricia Pavez, Barbara Valente, Pamela Donoso, Pablo M. Lavados.

**Resources:** Veronica V Olavarria, Valeska Vollrath, Pablo M. Lavados.

**Software:** Veronica V Olavarria, Paola Campodónico, Pablo M. Lavados.

**Supervision:** Veronica V Olavarria, Paula von Geldern, Carolina Velásquez, Patricia Pavez, Barbara Valente, Pamela Donoso, Pablo M. Lavados.

**Validation:** Veronica V Olavarria, Paola Campodónico, Valeska Vollrath, Paula von Geldern, Carolina Velásquez, Patricia Pavez, Barbara Valente, Pamela Donoso, Alexandra Ginesta, Pablo M. Lavados.

**Visualization:** Veronica V Olavarria, Pablo M. Lavados.

**Writing – original draft:** Veronica V Olavarria, Paula von Geldern, Carolina Velásquez, Patricia Pavez, Barbara Valente, Pamela Donoso, Gabriel Cavada, Pablo M. Lavados.

**Writing – review & editing:** Veronica V Olavarria, Paola Campodónico, Valeska Vollrath, Paula von Geldern, Carolina Velásquez, Patricia Pavez, Barbara Valente, Pamela Donoso, Alexandra Ginesta, Gabriel Cavada, Enrico Mazzon, Víctor Navia, Matías Guzmán, Pablo Brinck, Pablo M. Lavados.

## Supplementary Material

Supplemental Digital Content
