# Supplementary material for: Effects of an Avocado-based Mediterranean Diet on Serum Lipids for Secondary Prevention after Ischemic Stroke Trial (ADD-SPISE): Study protocol
Source: Medicine (Baltimore). 2021 Jun 18;100(24):e26425. doi: 10.1097/MD.0000000000026425 (PMC8213277; doi:10.1097/MD.0000000000026425)
Supplement: Supplemental Digital Content [file medi-100-e26425-s001.doc]

Supplemental digital content 1

14-point Mediterranean Diet Adherence Screener (MEDAS)

| **Item** | **Criteria for obtaining 1 point** |
| --- | --- |
| **1. Do you use olive oil as the principal source of fat for cooking?**  ¿Usa usted el aceite de oliva como principal grasa para cocinar? | Yes = 1 point |
| **2. How much olive oil do you consume per day (including that used in frying, meals eaten away from home, salads, etc.)? (1 tablespoon = 13.5g).**  ¿Cuánto aceite de oliva consume en total al día (incluyendo el usado para freír, comidas fuera de casa, ensaladas, etc.)? | 4 or more  tablespoons = 1 point |
| **3. How many servings of vegetables do you consume per day? (1 serving = 200g - side dishes count as ½ a serving, not including potatoes or sweetcorn).**  ¿Cuántas porciones de verduras u hortalizas consume al día? (las porciones o acompañamientos equivalen a 1/2 ración) 1 porción = 200g. | 2 or more (at  least 1 portion  raw or as salad) = 1 point |
| **4. How many pieces of fruit (including fresh-squeezed fruit juice) do you consume per day? (not including frozen or dried fruit).**  ¿Cuántas unidades de fruta (incluyendo las que utiliza para hacer jugo natural) consume al día? | 3 or more = 1 point |
| **5. How many servings of red meat, hamburger, or meat products (ham, sausage, etc.) do you consume per day? (1 serving = 100-150g)**  ¿Cuántas porciones de carnes rojas, hamburguesas, salchichas o embutidos consume al día? (porción: 100 - 150 g equivalente a la palma de la mano o 1 unidad de hamburguesa o vienesa) | Less than 1= 1 point |
| **6. How many servings of butter, margarine, or cream do you consume per day? (1 serving = 12g, 1 tablespoon).**  ¿Cuántas porciones de mantequilla, margarina o crema consume al día? (considerando una porción individual equivalente a 1 cucharada de sopera o 12 g) | Less than 1= 1 point |
| **7. How many sugar-sweetened beverages do you drink per day? (1 cup = 100 ml).**  ¿Cuántas bebidas carbonatadas y/o azucaradas (como refrescos, bebidas colas, tónicas) consume al día? | Less than 1 cup = 1 point |
| **8. How much wine do you drink per week? (1 glass = 125 ml)**  ¿Bebe usted vino? ¿Cuánto consume a la semana? | 7 or more  Glasses = 1 point |
| **9. How many servings of legumes do you consume per week? (1 serving = 150g) (including canned varieties).**  ¿Cuántas porciones de legumbres consume a la semana? (una porción equivale a un plato hondo o 150 g) | 3 or more = 1 point |
| **10. How many servings of fish or shellfish/seafood do you consume per week? *(1 serving = 100-150 g fish, or 4-5 pieces or 200 g shellfish).***  ¿Cuántas porciones de pescado o mariscos consume a la semana? (1 plato pieza o ración equivale a 1 palma de mano de pescado (100 – 150g) o 4-5 piezas de marisco(200g) ) | 3 or more = 1 point |
| **11. How many times per week do you consume commercial sweets or pastries (not homemade), such as cakes, cookies, biscuits, or custard?** ¿Cuántas veces consume productos de repostería comercial (no casera) como galletas, flanes, dulce o pasteles a la semana? | 2 or less = 1 point |
| **12. How many servings of nuts (including peanuts) do**  **you consume per week? (1 serving = 30g).**  ¿Cuántas veces consume frutos secos a la semana? Como nueces, almendras, maní, pistachos (una ración equivale a un puño de mano cerrada o 30 g) | 3 or more = 1 point |
| 13. **Do you prefer to eat chicken, turkey, or rabbit meat instead of beef, pork, hamburgers, or sausages?**  ¿Consume usted preferentemente carne de pollo, pavo en vez de vacuno, cerdo, hamburguesas o salchichas? (carne de pollo: 1 pieza o ración de 100 - 150 g) | Yes = 1 point |
| **14. How many times per week do you consume cooked vegetables, pasta, rice, or other dishes prepared with a sauce of tomato, garlic, onions or leeks sautéed in olive oil (sofrito)?**  ¿Cuántas veces a la semana consume vegetales, pasta, arroz u otros platos cocinados con un sofrito (de salsa de tomate, ajo, cebolla o puerro elaborada a fuego lento con aceite de oliva)? | 2 or more = 1 point |
| Total score: |  |

*Quantification of inflammatory markers:*

Blood samples are collected in tubes without anticoagulant. Aliquots of plasma are transferred to Eppendorf tubes and kept at -80 °C until the time of analysis. Quantification of Intercellular Adhesion Molecule 1 (ICAM), Vascular Cell Adhesion Molecule-1 (VCAM) and Interleukin 6 (IL6) are determined using ELISA kits of immunoassays (R&D System) following manufacturer instructions. Note that, ICAM, VCAM e IL6 follow the same analysis and they are worked separately. It employs the quantitative sandwich enzyme immunoassay technique follows by UV-visible absorption using a Synergy-4 from Biotek equipment. A monoclonal antibody specific for human IL-6, ICAM-1 and VCAM-1, respectively have been pre-coated onto each microplate. Standards and samples are pipetted into the wells and any IL-6 (ICAM-1 or VCAM-1) present is bound by the immobilized antibody. After washing away any unbound substances, an enzyme-linked polyclonal antibody specific for human IL-6 (ICAM-1 or VCAM-1) is added to the wells. Following a wash to remove any unbound antibody-enzyme reagent, a substrate solution is added to the wells and color develops in proportion to the amount of IL-6 (ICAM-1 or VCAM-1) bound in the initial step. The color development is stopped and the intensity of the color is measured at 450 nm (specific wavelength) with correction to 540 nm or 570 nm (wavelengths for plastic plate). Note that, a duplicate of readings for each standard is subtracted by the average zero standard given by the calibrator diluent alone to build the standard curve.

*Human IL-6 Immunoassay* create a standard curve by reducing the data using computer software capable of generating a four parameters logistic (4-PL) curve fit. If samples have been diluted, the concentration read from the standard curve is multiplied by the dilution factor. The minimum detectable dose of human IL-6 is less than 0.70 pg/mL. The IL-6 assay has a reportable range from 0.7 to 12.5 pg/mL.

*Human ICAM-1/CD54 Allele-specific Immunoassay.* Plasma samples require a 20-fold dilution and at the end the concentration read from the standard curve must be multiplied by the dilution factor. The standard curve is performed by a computer software capable of generating a four parameters logistic (4-PL) curve fit. The minimum detectable dose of human ICAM-1 is ranged from 0.096 to 0.254 ng/mL. The mean minimum detectable dose was 0.096 ng/mL. The ICAM-1 assay has a reportable range from 106 to 337 ng/mL with a standard deviation of 41.6 ng/mL.

*Human VCAM-1/CD106 Immunoassay.* Plasma samples require a 20-fold dilution. At the end, the concentration read from the standard curve must be multiplied by the dilution factor. The standard curve is executed by a computer software capable of generating a log/log curve-fit. The minimum detectable dose of human VCAM-1 is ranged from 0.17 to 1.26 ng/mL. The mean minimum detectable dose was 0.6 ng/mL. The ICAM-1 assay has a reportable range from 301 to 875 ng/mL with a standard deviation of 149.3 ng/mL.

Finally, each measure of sample is in duplicate and if sample results fall outside acceptable ranges for their calibration curve; the samples containing a known concentration of IL-6, VCAM-1 and ICAM-1 must be diluted with saline and the resulting samples should be analyzed. A detailed information about the assay procedures can be found in each data sheet of R&D System available on: [www.rndsystems.com](http://www.rndsystems.com) and references therein there.

*Apo lipoprotein A-1 (Apo A-1) and Apo lipoprotein B (Apo B)* are analysed using turbidimetry test reagents kits (Biosystems®) in an automatic analyser (Selectra XL-Pro Vitalab®) from serum samples. The ApoA-1 and ApoB assays are an immunoturbidimetric procedures that measures increasing sample turbidity caused by the formation of insoluble immune complexes when antibody to ApoA-1 and ApoB, respectively are added to the sample. The ApoA-1/Apo B concentrations are measured as a function of turbidity. The methodology used is immunoturbidimetric. A multi-point calibration (Linear) curve is generated using ApoA-1/Apo B Calibrator, respectively. Each calibration curve is verified with at least three measurements of control according to the established quality control requirements for our laboratory. If control results fall outside acceptable ranges, recalibration may be necessary. Randox HN1530 control is used to verify the immunoturbidimetric method and the calibration curve. The ApoA-1 has a range between 95.9 to 138 mg/dL with a target located at 117 mg/dL and the range for Apo B is located between 51 to 73.4 mg/dl with a target in 62.2 mg/dL. The ApoA-1 assay reportable range is from 1.9 to 250 mg/dL; with a limit of quantification of 1.9 mg/dL. The ApoB assay reportable range is from 1.5 to 300 mg/dL; with a limit of quantification of 1.5 mg/dL. Finally, if the results fall outside acceptable ranges for a calibration curve; the samples containing a known concentration of ApoA-1/Apo B are diluted with saline and the resulting samples are be analyzed.
